# Development of a new set of reference genes for normalization of real-time RT-PCR data of porcine backfat and *longissimus dorsi *muscle, and evaluation with *PPARGC1A*

**DOI:** 10.1186/1472-6750-6-41

**Published:** 2006-10-09

**Authors:** Tim Erkens, Mario Van Poucke, Jo Vandesompele, Karen Goossens, Alex Van Zeveren, Luc J Peelman

**Affiliations:** 1 Department of Nutrition, Genetics and Ethology, Faculty of Veterinary Medicine, Ghent University, Heidestraat 19, 9820 Merelbeke, Belgium; 2 Centre for Medical Genetics, Ghent University Hospital, De Pintelaan 185, 9000 Ghent, Belgium

## Abstract

**Background:**

An essential part of using real-time RT-PCR is that expression results have to be normalized before any conclusions can be drawn. This can be done by using one or multiple, validated reference genes, depending on the desired accuracy of the results. In the pig however, very little information is available on the expression stability of reference genes. The aim of this study was therefore to develop a new set of reference genes which can be used for normalization of mRNA expression data of genes expressed in porcine backfat and *longissimus dorsi *muscle, both representing an economically important part of a pig's carcass. Because of its multiple functions in fat metabolism and muscle fibre type composition, *peroxisome proliferative activated receptor γ coactivator 1α *(*PPARGC1A*) is a very interesting candidate gene for meat quality, and was an ideal gene to evaluate our developed set of reference genes for normalization of mRNA expression data of both tissue types.

**Results:**

The mRNA expression stability of 10 reference genes was determined. The expression of *RPL13A *and *SDHA *appeared to be highly unstable. After normalization to the geometric mean of the three most stably expressed reference genes (*ACTB*, *TBP *and *TOP2B*), the results not only showed that the mRNA expression of *PPARGC1A *was significantly higher in each of the *longissimus dorsi *muscle samples than in backfat (P < 0.05), but also that the expression was significantly higher in the most cranial of the three muscle samples (P < 0.05).

**Conclusion:**

This study provides a new set of reference genes (*ACTB*, *TBP *and *TOP2B*) suitable for normalization of real-time RT-PCR data of backfat and *longissimus dorsi *muscle in the pig. The obtained *PPARGC1A *expression results, after application of this set of reference genes, are a first step in unravelling the PPARGC1A expression pattern in the pig and provide a basis for possible selection towards improved meat quality while maintaining a lean carcass.

## Background

Because of consumers' demands, the emphasis of modern day porcine breeding programmes and selection criteria is gradually shifting towards a higher meat quality, instead of the more classical focus of just selecting for a lean carcass and high growth rate [1]. Two strongly genetically determined factors influencing meat quality are the amount of intramuscular fat (IMF) and muscle fibre type composition [2]. The first factor has a large impact on the taste of the meat and its deposition is strongly influenced by the surrounding muscle [3]. Peri- and post-mortem biochemical processes in muscle and meat are strongly influenced by muscle fibre type composition and in their turn influence meat tenderness [4]. The fibre type composition depends on the location and use of a muscle. Muscles used for posture are more oxidative, while more active muscles contain more glycolytic fibres [2]. In general, meat quality increases if more oxidative fibres and less glycolytic fibres are present, but the influence of fat distribution and fibre type composition on meat quality also varies between different breeds [1,5,6].

Unfortunately the selection of leaner pigs was often accompanied by a decrease in IMF and a lower percentage of oxidative fibres, resulting in a decrease of meat quality [7,8]. To understand the complex relationship between all the traits affecting meat quality, it is important to trace the responsible genes. One particularly interesting gene in this regard is the *peroxisome proliferative activated receptor γ coactivator 1α *(*PPARGC1A*), a coactivator that influences the expression of many genes through a whole range of nuclear hormone receptors and other transcription factors, like the peroxisome proliferative activated receptors (PPAR α, β, γ), nuclear respiratory factors and the thyroid hormone receptor [9,10]. PPARGC1A plays an important role in adipogenesis and adipocyte differentiation [11], mitochondrial biogenesis and respiration [9,12], and hepatic gluconeogenesis [13]. This means that the gene is involved in adaptive thermogenesis, fat metabolism and energy homeostasis. PPARGC1A can be induced by cold and exercise, it has been proven to function as a natural protection against obesity in brown fat of mice by producing heat and it is especially expressed in tissues with a high energy demand, like muscle and brown fat [9,10,14]. The expression is higher in muscles that contain more oxidative fibres and it has been shown that PPARGC1A is an important factor in determining fibre type in that it enhances the number of oxidative muscle fibres [15,16]. Also, other candidate genes for meat quality like *glucose transporter 4 *(*GLUT4*) are regulated by PPARGC1A, making it an even more interesting gene [17]. All these functions and the fact that already a quantitative trait locus for leaf fat weight in the pig has been located in the chromosomal region to which *PPARGC1A *was mapped [18], illustrate that *PPARGC1A *is a very promising candidate gene with regard to meat quality.

In humans, *PPARGC1A *has been thoroughly investigated because of its presumed role in the obesity pandemic and correlated diseases like type II diabetes mellitus and cardiovascular complications, especially in search of possible future therapies [19-21]. In the pig however, very little is known about *PPARGC1A *although it has a great economic potential and, because of the strong genetical resemblance between pig and man, the information could be useful in human research as well [22,23]. Jacobs *et al*. [18] already described the porcine *PPARGC1A *coding sequence and discovered several polymorphisms, of which at least one was significantly associated with backfat.

To study mRNA expression levels, one needs a set of carefully selected and validated reference genes (also called housekeeping genes) for normalization purposes. In the pig however, virtually no information is available on reference genes. In this study, we therefore designed assays to measure the mRNA expression levels of several candidate reference genes, followed by determination of their expression stability and suitability for normalization purposes in the tissues of interest. The first aim of this study was to develop a set of reference genes that can be used for normalizing real-time RT-PCR mRNA expression data from backfat and *longissimus dorsi *muscle of the pig. These two tissues comprise an important part of the carcass and have quite opposite demands regarding fat content, as explained above. If the regulation of PPARGC1A expression proves to be different in these tissues, unravelling the mechanism can possibly enable future selection towards one feature without influencing the other. Therefore the second aim of our study was to assess whether *PPARGC1A *mRNA expression differs between backfat and several *longissimus dorsi *muscle locations.

## Results

### Reference gene expression and selection

**Figure 1:**
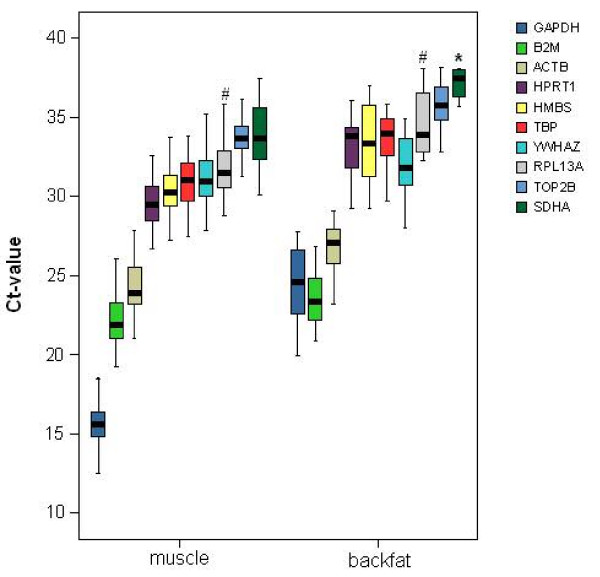
**Ct-value range of the reference genes**. The thick, black line is the median. The coloured box represents 50% of the measurements for a gene. #: depending on the animal, RPL13A was detected either in both muscle and fat, only in muscle, or was not detected at all. *: in most of the backfat samples no SDHA was detected.

**Figure 2:**
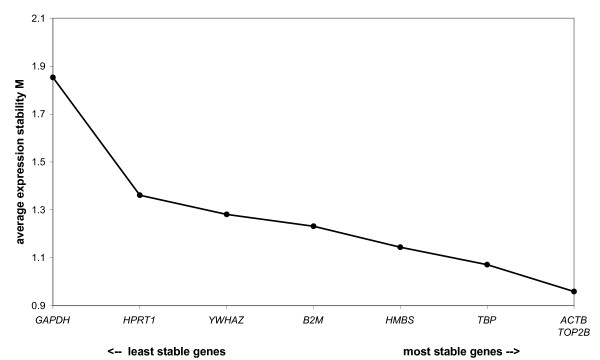
Reference gene mRNA expression stability according to geNorm.

The cycle threshold value (Ct-value: the fractional PCR cycle at which the fluorescent signal significantly rises above the background signal) range of the reference genes is shown in Figure 1 and demonstrates that their expression levels can vary considerably. Backfat samples sometimes showed a minimal *SDHA *mRNA expression (Ct > 36), but for most of these samples no *SDHA *could be detected in our experimental setup. The expression pattern of *RPL13A *varied between animals: either there was expression in all 4 tissue samples of the same animal, or no Ct-values could be measured, or expression could only be detected in muscle samples but not in fat. For the other 8 reference genes, Ct-values were detected in all 4 tissue types and their raw data were analyzed using the geNorm algorithm [24]. The stepwise exclusion of the reference gene with the least stable expression showed that *ACTB *and *TOP2B *were the most stably expressed reference genes in the analyzed samples (Figure 2). Of note, the conventionally used reference gene *GAPDH *proved to be quite unstable in our tissue samples, further underscoring the need for reference gene evaluation. GeNorm indicated that normalization using a set of the 3 most stably expressed reference genes (*ACTB*, *TOP2B *and *TBP*) would provide reliable results for mRNA expression analysis in backfat and *longissimus dorsi *muscle.

### PPARGC1A expression

**Figure 3:**
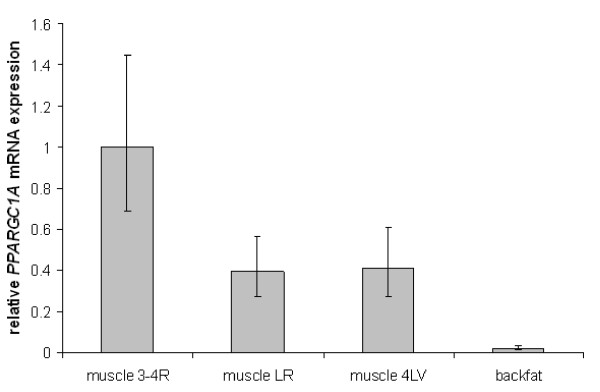
**Relative, normalized and rescaled *PPARGC1A *mRNA expression of 20 pigs**. Muscle 3-4R are the *longissimus dorsi *samples taken near 3^rd ^or 4^th ^rib, muscle LR is taken near the last rib and muscle 4LV near the 4^th ^lumbar vertebra. Error bars represent the 95% confidence interval.

Figure 3 visualizes the *PPARGC1A *expression differences between the rescaled, normalized data of the 20 pigs used for determining the reference gene stability. The 95% confidence intervals (represented by the error bars) clearly indicate that the mRNA expression of *PPARGC1A *is significantly higher (P < 0.05) in the 3 muscle samples than in backfat of the pig. It also shows that the expression in the *longissimus dorsi *muscle sample taken near the 3^rd ^or 4^th ^rib is significantly higher (P < 0.05) than in the other 2 muscle samples. No significant *PPARGC1A *expression difference was found between these 2 other muscle samples.

The same analysis was performed on the data obtained from the larger independent group of 30 pigs and showed exactly the same significant differences in *PPARGC1A *expression (See additional file 1: AddFileRelativeExpression30pigs.doc).

## Discussion

Real-time RT-PCR is a sensitive and accurate technique for measuring gene expression [25], but to be able to compare mRNA expression across samples, it is essential to correct for variables such as differences in the amount of starting material in the reaction and enzyme efficiency. This normalization can be performed by using internal reference genes. Contrary to original expectations, it has been proven that their expression level can vary extensively with tissue type and (experimental) conditions, and that their use needs to be validated for each type of tissue and every experimental setup [26-28]. Furthermore Vandesompele *et al*. [24] showed that more than one reference gene should be used for accurate normalization of expression data.

In the pig however, very little is known about the expression of reference genes, certainly in combination with real-time RT-PCR. Analysis of the expression stability of the 10 chosen reference genes showed that *RPL13A *and *SDHA *cannot be used for normalization of *longissimus dorsi *and backfat data. The expression of *RPL13A*, a component of the 60S ribosomal subunit, varied per animal. As far as we know, this is the first time such an expression pattern is described for this reference gene, and could reflect a large difference in basic transcriptional activity depending on the animal and/or tissue used. Because of the importance of a correct protein synthesis mechanism, it is likely that for the samples in which no *RPL13A *was detected, its function was (partly) taken over by another ribosomal protein [29-31], or that an alternative splice product exists to which the used primer(s) could not anneal [32,33]. Another possible explanation for this variability is technical variation, because the assay could have been at the limit of its sensitivity. *SDHA*, an enzyme involved in energy production (Krebs cycle and respiratory chain) [34], could not be detected in backfat samples, except for a very weak signal in a few pigs. This could indicate SDHA is present in much lower concentrations in backfat compared to *longissimus dorsi *muscle, which agrees with one of the main functions of white adipose tissue, storing energy. But from the data presented here no definitive conclusions can be drawn regarding the cause of this variability. 

As mentioned before, expression results are considerably more reliable if they are normalized using the geometric mean of multiple reference genes [24]. GeNorm analysis indicated that addition of the 3^rd ^reference gene to the normalization factor had the largest impact on reducing variability. Though variability further decreased by including a 4^th ^and 5^th ^gene, the decrease is minimal and does not outweigh the practical considerations accompanying the inclusion of more genes. Therefore, the use of the geometric mean of the 3 most stably expressed reference genes (*ACTB*, *TOP2B *and *TBP*; Figure 2) provides reliable results for backfat and *longissimus dorsi *mRNA expression comparison in the pig.

Although it has been investigated which tissues express *PPARGC1A *and what the effects of sequence polymorphisms are [16,18,35], little is known in the pig about its relative mRNA expression when comparing tissues to one another. Because this knowledge is essential in researching differences in *PPARGC1A *regulation in view of selection for improved meat quality, we compared its mRNA expression in 2 economically important tissue types. Our results not only clearly indicate that the *PPARGC1A *mRNA expression is significantly higher in *longissimus dorsi *muscle than in backfat (P < 0.05), but also that its expression is significantly higher in the most cranial of the 3 muscle samples (P < 0.05; Figure 3). The PCR efficiencies of the standard curves indicated that the lower mRNA expression of *PPARGC1A *in backfat was not due to inhibition. Nevertheless, if there would be any inhibition present, this should be corrected for by normalization. These results correspond with findings in humans where *PPARGC1A *mRNA expression in *vastus lateralis *muscle was clearly higher than in subcutaneous fat [36], and are similar to studies in the pig on the mRNA expression of *PPARγ *(one of the transcription factors coactivated by PPARGC1A) which showed variation between visceral and subcutaneous fat [37]. In a study on the effect of exercise on PPARGC1A protein expression in rats, differences were not only found between various skeletal muscles, but also basal protein expression in a control group differed between the more oxidative and the more glycolytic part of the *gastrocnemius *muscle [38]. Keeping this in mind, a possible explanation for the higher mRNA expression in the most cranial *longissimus dorsi *sample could be that because of its location, it has other energy demands and fibre type composition compared to more caudal parts of the muscle. Our findings could indicate a tissue and location specific regulation of *PPARGC1A *expression correlating with the functions of PPARGC1A in *longissimus dorsi *muscle and backfat [20]. But on the other hand mRNA expression differences do not necessarily translate into protein expression differences [39].

## Conclusion

The data from this study not only present a newly developed set of reference genes for normalization of mRNA expression data from porcine backfat and *longissimus dorsi *muscle, but also prove that significant differences exist in *PPARGC1A *mRNA expression between and within (for muscle) these economically important tissues, providing a basis for possible selection towards improved meat quality while maintaining a lean carcass.

## Methods

### Sample collection and cDNA synthesis

In a commercial pig slaughterhouse 4 samples were carefully collected from each of 50 cleaved pigs: a sample of the *longissimus dorsi *muscle near the 3^rd ^or 4^th ^rib, a sample of the *longissimus dorsi *muscle near the last rib, a *longissimus dorsi *sample near the 4^th ^lumbar vertebra, and a sample of backfat. The samples (thickness 0.3 – 0.4 cm) were immediately submerged in RNA*later *(Sigma-Aldrich) for RNA preservation, after which they were crushed to powder with liquid nitrogen, subdivided per 80–100 mg and stored at -80°C, until total RNA extraction with TRIR (ABgene) according to the manufacturer's protocol. During the next step contaminating DNA was degraded by treating each sample with RQ1 RNase-free DNase (Promega) according to the instructions manual, followed by a spin-column purification (Microcon YM-100, Millipore). During optimization of the RNA extraction protocol, RNA integrity was verified by loading RNA onto a 0.8% agarose gel and evaluating the 28S and 18S ribosomal RNA bands. Backfat RNA showed slightly more degradation compared to muscle RNA, but this was no problem because of the small size of the amplicons used. After verification of the absence of any DNA contamination by means of a minus reverse transcription (RT) control PCR (which included a positive porcine genomic DNA control and a no-template control) using the *YWHAZ *primers (Table 1), the purity and RNA concentration was measured with a BioPhotometer (Eppendorf). The OD 260/280 ratio of the samples ranged between 1.75 – 2.15. Concentrations for muscle samples ranged between 80–200 ng/μl (total yield 1.76–4.40 μg RNA) and for fat samples between 40–100 ng/μl (total yield 0.88–2.20 μg RNA). In this way approximately 1 μg of RNA from each sample could be converted to cDNA in the subsequent 20 μl RT reaction with the iScript cDNA synthesis kit (Bio-Rad), which contains both oligo dT and random primers. The cDNA was diluted 10 times with Tris-HCl (pH 8, 10 mM) before verification of the RT reaction through a control PCR using 2.5 μl cDNA and the same *YWHAZ *primers as mentioned above (Table 1). Each PCR also included a negative control to check for DNA contamination.

### Primers

From literature 10 reference genes were selected (Table 2) [24,40], belonging to different functional classes to minimize the chance of coregulation. Primers for *TOP2B *were used from Van Poucke *et al*. [40]. The NCBI [41] and Ensembl [42] databases were used to search for available porcine sequences of the other 9 reference genes in order to design primers with Primer3 [43], taking into account the possible secondary structures of the amplicon (Mfold) [44] and the amplicon specificity of the primers (Blast) [45]. Whenever possible, intron-spanning primers were selected as an extra control to be able to distinguish between cDNA and contaminating genomic DNA. Primer conditions were optimized by determining the optimal annealing temperature and primer concentration, and amplicons were verified by sequencing with an ALFexpress (Amersham Biosciences). The primer pair used for measuring mRNA expression of *PPARGC1A *was selected from Jacobs *et al*. [18]. Table 1 summarizes the information on the primers, including their GenBank accession numbers.

### Real-time PCR assays

Real-time PCR was conducted on the iCycler iQ Real-Time PCR Detection System (Bio-Rad), each reaction consisting of 7.5 μl Platinum SYBR Green qPCR SuperMix UDG (uracil-N-glycosylase; Invitrogen) spiked with 0.15 pmole fluorescein calibration dye (Bio-Rad), 2.5 μl cDNA, the optimized amount of primer and supplemented with water (Molecular Biology Grade, Eppendorf) to a total volume of 15 μl. The real-time PCR program started with a 2 minute UDG incubation step at 50°C, followed by a 3 minute denaturation at 95°C, during which the hot start platinum *Taq *DNA polymerase was fully activated. This was followed by 40 cycles of 15 seconds of denaturation at 95°C and 30 seconds of annealing/elongation at the optimal annealing temperature (Ta) for each specific primer (Table 1), during which fluorescence was measured. Next a melting curve was constructed by increasing the temperature from 70 to 95°C in sequential steps of 0.5°C for 10 seconds, at which fluorescence was measured. This allowed the verification of the presence of one gene-specific peak and the absence of primer dimer peaks, which also give a fluorescent signal and influence PCR efficiency. During optimization all PCR products were loaded onto a 2% agarose gel (Gentaur) for verification. A 10-fold dilution series of cDNA was included in each run to determine PCR efficiency by constructing a relative standard curve. PCR efficiencies were consistently > 92% and were used to convert the Ct-values into raw data (not yet normalized, relative quantities). All experiments contained a negative control and samples were analyzed in 2 independent runs.

### Reference gene selection

To determine the mRNA expression stability of the 10 reference genes, their expression in the 4 tissue samples (biological replicates) of 20 randomly selected animals was measured using real-time PCR, as described above. This means that for each reference gene 80 reactions were performed in duplicate (technical replicates), in 2 separate runs. To correct for technical inter-run variation between replicated reactions of the same sample measured in different runs, the data from these 2 runs were calibrated by calculating the average Ct-value over all the samples in each run and subtracting the difference between these 2 averages from each individual sample in the run with the highest average Ct-value. After this calibration step, the average Ct-value of each duplicate reaction was converted to relative quantities and these were analyzed using the geNorm algorithm, which is based on the principle that the expression ratio of 2 ideal reference genes should be identical in all samples [24]. Using this algorithm, the most stably expressed reference genes and their optimal number for normalization were determined.

The same procedure of real-time PCR, calibration and conversion to relative quantities was repeated for the 4 tissue samples of the remaining 30 animals, performed with only the 3 most stably expressed reference genes as determined by geNorm.

### PPARGC1A mRNA expression and data processing

Real-time PCR was conducted with the primers for *PPARGC1A *on the 4 samples of all 50 animals, after which the mRNA expression data were calibrated and converted into raw data in the same manner as described above for the reference genes. Then for each animal the geometric mean of the raw expression data of the 3 most stably expressed reference genes (i.e. normalization factor, NF) was calculated and used for subsequent normalization, dividing the raw *PPARGC1A *mRNA expression data by the NF. These normalized expression levels of *PPARGC1A *were then converted into logarithmic values, the average per sample type and the 95% confidence interval was calculated, these in their turn were converted into linear rescaled values and eventually plotted in a graph (Figure 3).

## Authors' contributions

TE performed all the experimental procedures and was the primary author of the manuscript. MVP and KG participated in the study design and provided real-time support. JV provided expert input in data analysis. AVZ and LJP participated in the design of the project, helped to draft the manuscript and supervised the study. All authors read and approved the final manuscript.

## Supplementary Material

Additional file 1 Relative, normalized and rescaled *PPARGC1A *mRNA expression of 30 pigs. Muscle 3-4R are the *longissimus dorsi *samples taken near 3^rd ^or 4^th ^rib, muscle LR is taken near the last rib and muscle 4LV near the 4^th ^lumbar vertebra. Error bars represent the 95% confidence interval. Click here for file

## Figures and Tables

**Table 1:** Information on the primers used for real-time PCR

Gene	Primer sequence (5'→3')	Amplicon length	Ta	GenBank accession number or reference
*ACTB*	TCTGGCACCACACCTTCT TGATCTGGGTCATCTTCTCAC	114 bp	60°C	[GenBank:DQ178122]
*B2M*	AAACGGAAAGCCAAATTACC ATCCACAGCGTTAGGAGTGA	178 bp	60°C	[GenBank:DQ178123]
*GAPDH*	ACTCACTCTTCTACCTTTGATGCT TGTTGCTGTAGCCAAATTCA	100 bp	57°C	[GenBank:DQ178124]
*HMBS*	CTGTTTACCAAGGAGCTGGAAC TGAAGCCAGGAGGAAGCA	100 bp	59°C	[GenBank:DQ178125]
*HPRT1*	CCGAGGATTTGGAAAAGGT CTATTTCTGTTCAGTGCTTTGATGT	181 bp	60°C	[GenBank:DQ178126]
*RPL13A*	AGTTAAAGTACCTGGCCTTCCT TGGCCTCTCTTGGTCTTG	136 bp	59°C	[GenBank:DQ178127]
*SDHA*	GAACCGAAGATGGCAAGA CAGGAGATCCAAGGCAAA	191 bp	58°C	[GenBank:DQ178128]
*TBP*	GATGGACGTTCGGTTTAGG AGCAGCACAGTACGAGCAA	124 bp	59°C	[GenBank:DQ178129]
*TOP2B*	AACTGGATGATGCTAATGATGCT TGGAAAAACTCCGTATCTGTCTC	137 bp	60°C	[40]
*YWHAZ*	ATGCAACCAACACATCCTATC GCATTATTAGCGTGCTGTCTT	178 bp	60°C	[GenBank:DQ178130]
*PPARGC1A*	CCTGCATGAGTGTGTGCTCT CTCAGAGTCCTGGTTGCACA	107 bp	59°C	[18]

**Table 2:** Full reference gene names

	Full gene name
*ACTB*	*beta actin*
*B2M*	*beta-2-microglobulin*
*GAPDH*	*glyceraldehyde-3-phosphate dehydrogenase*
*HMBS*	*hydroxymethylbilane synthase*
*HPRT1*	*hypoxanthine phosphoribosyltransferase 1*
*RPL13A*	*ribosomal protein L13a*
*SDHA*	*succinate dehydrogenase complex, subunit A*
*TBP*	*TATA box binding protein*
*TOP2B*	*topoisomerase II beta*
*YWHAZ*	*tyrosine 3-monooxygenase/tryptophan 5-monooxygenase activation protein, zeta polypeptide*
