# Supplementary material for: Development of a new set of reference genes for normalization of real-time RT-PCR data of porcine backfat and longissimus dorsi muscle, and evaluation with PPARGC1A
Source: BMC Biotechnol. 2006 Oct 9;6:41. doi: 10.1186/1472-6750-6-41 (PMC1609116; doi:10.1186/1472-6750-6-41)
Supplement: Supplementary file 1 [file 1472-6750-6-41-S1.doc]

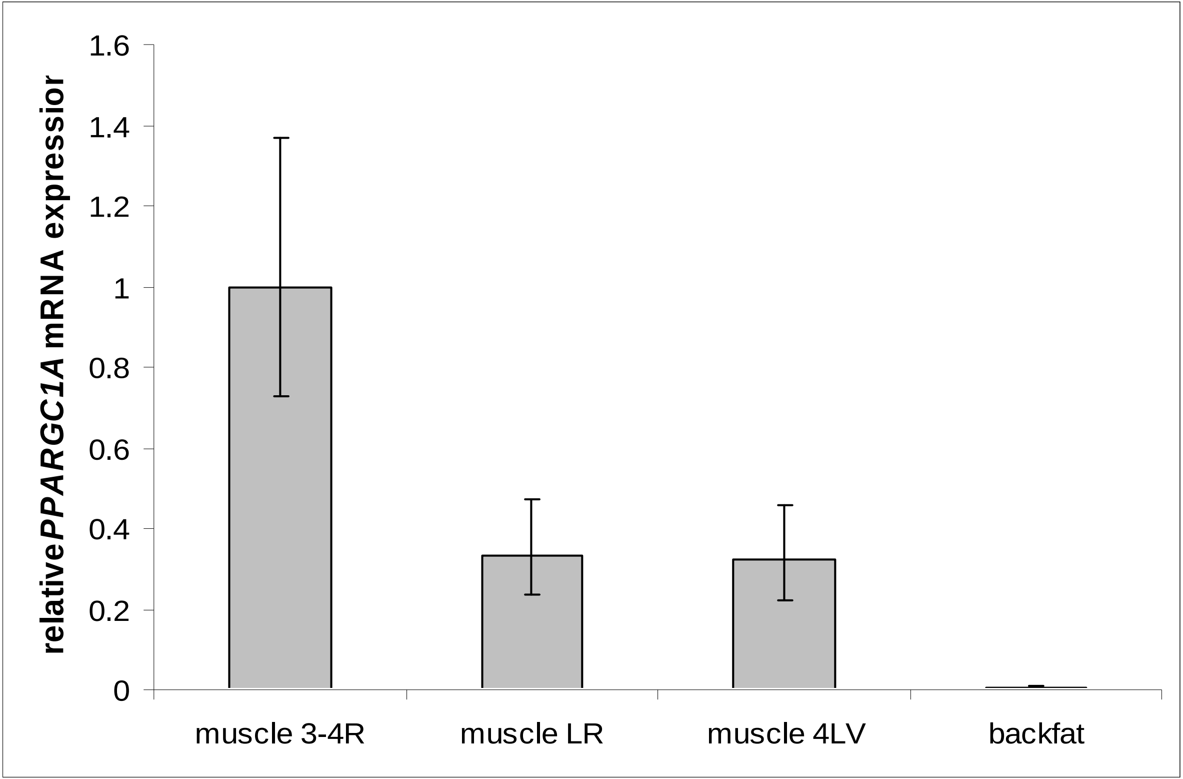

relative *PPARGC1A* mRNA expression
1.6
1.4
1.2
1
0.8
0.6
0.4
0.2
0
muscle 3-4R
muscle LR
muscle 4LV
backfat
